# How the chromatin landscape influences nuclear morphology

**DOI:** 10.3389/fcell.2025.1634252

**Published:** 2025-07-03

**Authors:** Sourabh Sengupta, Haritha Prabha, Daniel L. Levy

**Affiliations:** ^1^ Department of Cell Biology, University of Texas Southwestern Medical Center, Dallas, TX, United States; ^2^ Department of Molecular Biology, University of Wyoming, Laramie, WY, United States

**Keywords:** nuclear size, nuclear shape, chromatin structure, chromatin modifications, cancer, epigenetics, *Xenopus* egg extract, microscopy

## Abstract

Nuclear morphology is a defining cellular feature, differing based on cell type, tissue type, and species. In healthy cells, nuclear morphology is generally tightly regulated and maintained; however, dynamic changes in nuclear morphology are observed under certain conditions, for instance in early embryos and in some immune cells. Deviations in normal nuclear morphology are linked to numerous diseases, including most cancers and premature aging syndromes. Many regulators of nuclear morphology have been identified, encompassing both intranuclear, cytoplasmic, and extracellular factors. Of note, recent studies have converged on chromatin and chromatin-associated proteins as key determinants of nuclear morphology and dynamics. In this review we discuss how the chromatin landscape regulates nuclear morphology in both normal and diseased cellular states. Additionally, we highlight emerging technologies that promise to bridge critical gaps in our understanding of nuclear morphology, including new approaches to probe nuclear structure and the use of synthetic cells.

## 1 Introduction

Cells vary substantially in size and morphology, ranging from yeast cells, which have a diameter of 3 μm, to nerve cells in the neck of the giraffe, which can be 3 m in length. A fundamental question is how organelle size and shape are tuned to support the structure and function of such diverse cell types. The regulation of nuclear size and shape is one area of particular interest (Marshall, 2002; Levy and Heald, 2012; Edens and Levy, 2014; Jevtic et al., 2015).

**FIGURE 1:**
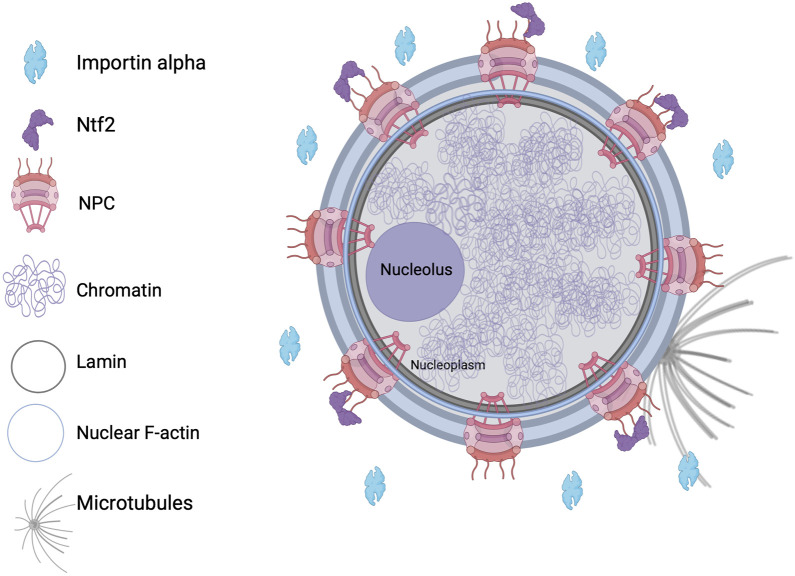
Nuclear structure and known regulators of nuclear morphology. NPC, nuclear pore complex.

In eukaryotic cells, the nucleus is comprised of a double-membrane nuclear envelope (NE) that encloses the DNA and is often continuous with the endoplasmic reticulum. Nucleoporins are the proteins that make up nuclear pore complexes (NPCs) which span the NE, traversing through the inner and outer nuclear membranes (Alber et al., 2007; Fernandez-Martinez and Rout, 2009). Selective transport of molecules occurs through these NPCs (D'Angelo and Hetzer, 2008; Lin and Hoelz, 2019). The nuclear lamina is a meshwork that lines the nucleoplasmic face of the inner nuclear membrane and is composed of intermediate filament lamin proteins and additional interacting proteins, providing mechanical support and structural organization (Figure 1).

The maintenance of organelle shape and size is likely critical for cellular function. Under physiological conditions, nuclear sizes typically scale with cell sizeto maintain a constant nuclear-to-cytoplasmic ratio (Conklin, 1912; Gregory, 2005; Jorgensen et al., 2007; Neumann and Nurse, 2007; Hara and Kimura, 2009; Jevtic and Levy, 2015; Vukovic et al., 2016; Cantwell and Nurse, 2019; Sengupta et al., 2025). With respect to shape, nuclei are generally roughly spherical, although there are variations based on cell type, species, and differentiation status. Important questions in cell biology relate to the mechanisms responsible for the regulation of nuclear morphology.

Human granulocytes exhibit multi-lobed nuclei, connected by short channels of nucleoplasm (Skinner and Johnson, 2017). Spindly-shaped or fusiform nuclei are commonly seen in human fibrocytes and syncytial endosperm of flowering plants like *Arabidopsis thaliana* (Skinner and Johnson, 2017). Pathological conditions, such as cancers and laminopathies, are associated with significant alterations in nuclear morphology. Mutations in lamin A are known to cause Hutchinson-Gilford progeria syndrome, which is characterized by dysmorphic nuclei (Scaffidi and Misteli, 2006). Enlarged nuclei are observed in various forms of cancer. Furthermore, lobulated nuclei are characteristic of adenocarcinomas, and nuclei with grooves and clefts are seen in thyroid cancers (Zink et al., 2004). These anomalies in nuclear morphologies are often used by clinicians for diagnostic purposes. Nuclear inclusions and abnormal nuclei are also associated with neurodegenerative disease (Woulfe, 2008). Importantly, biological processes, including gene expression and cell migration, can be influenced by nuclear morphology. For example, preventing dynamic changes in nuclear morphology impedes the transition of cells to S phase (Aureille et al., 2019). Cells with altered nuclear elasticity due to changes in lamin A/C levels exhibit altered migration capabilities (Bell and Lammerding, 2016).

**FIGURE 2:**
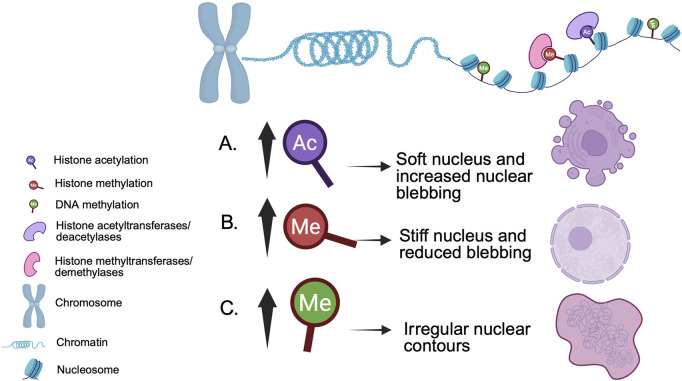
Example epigenetic modifications and their impact on nuclear morphology. **(A)** Nuclear softening and blebs caused by increased histone acetylation are reversed by **(B)** increased histone methylation (Stephens et al., 2018). **(C)** Increased DNA methylation is associated with irregularly shaped nuclei (de Capoa et al., 1996; Rougier et al., 1998).

In eukaryotes, genomic DNA is wrapped around histone octamers, comprising two copies each of the core histones H2A, H2B, H3, and H4, along with the linker histone H1. This facilitates the formation of highly condensed chromatin which, along with RNAs and other proteins, enables the packaging of DNA within the nucleus (Luger et al., 2012). Chromatin structure can be broadly divided into two types: heterochromatin which is more condensed and inhibitory for DNA metabolic processes and euchromatin which is more open and accessible to DNA-binding factors. The dynamism between euchromatin and heterochromatin is regulated by ATP-dependent chromatin remodelers, post-translational histone modifications like methylation, acetylation, and phosphorylation, and direct DNA methylation (Suganuma and Workman, 2011; Nodelman and Bowman, 2021; Mattei et al., 2022) (Figure 2). Of note, aberrations in these processes are the etiology of several diseases and disorders. Altered promoter methylation leads to aberrant gene expression in neurodevelopmental disorders like Parkinson’s, Alzheimer’s, and Huntington’s disease (Lu et al., 2013). Gain and loss of function mutations in histone modifiers are observed in various forms of cancer, such as lung squamous cell carcinoma (Brennan et al., 2017; Papillon-Cavanagh et al., 2017; Farhangdoost et al., 2021), clear cell renal carcinoma (Abuzeid et al., 1987), myeloid neoplasms, breast and prostate cancer (Varambally et al., 2002), and others (Janssen and Lorincz, 2022). Chromatin is the major occupant of the nucleus and, as such, it is perhaps not surprising that the chromatin landscape can modulate nuclear morphology. In this review, we first provide a broad overview of known regulators of nuclear morphology and then focus on more recent studies that highlight the roles of chromatin structure and factors in regulating nuclear morphology and, in turn, cellular and nuclear function.

## 2 Regulators of nuclear morphology

An important determinant of nuclear morphology is the nuclear lamina (Figure 1). The nuclear lamina contributes to the ability of the nucleus to respond to mechanical forces and to withstand intracellular and extracellular forces to avoid catastrophe (Swift et al., 2013; Swift and Discher, 2014; Morival et al., 2025). Lamin A/C restricts nuclear deformation while facilitating movement through narrow channels with smooth surfaces. Experiments with fibroblasts cultured on arrays of fibronectin-coated micropost barriers that mimic collagen fiber bundles showed that lamin A/C facilitates nuclear passage between slender obstacles. Nuclei containing lamin A/C preserve their oval shape despite local indentations. Conversely, nuclei deficient in lamin A/C experience severe distortion and become entangled around obstacles, impeding movement. This supports a model where the nucleus deforms like a droplet, with lamin A/C providing surface tension that allows for local invaginations that enable forward movement while maintaining overall shape (Katiyar et al., 2022).

Nuclei formed in *Xenopus laevis* egg extracts in the presence of dynamic F-actin exhibit a bilobed shape, with distinct membrane compositions in each lobe and F-actin concentrated at the inner nuclear envelope (Figure 1). Adding lamin A, not present in *Xenopus* eggs, results in more spherical nuclei. This indicates that a balance of forces exerted by nuclear F-actin and lamin A influences nuclear shape. Nuclear F-actin filaments, nucleated by formins, are thought to exert outward forces that alter nuclear morphology unless counterbalanced by lamin A (Mishra and Levy, 2022a). Microtubules can also influence nuclear morphology, for instance short term proteasome inhibition causes microtubule-mediated NE deformation independently of nuclear import (Sengupta et al., 2025). Nuclear lamin concentration can influence nuclear growth and size, with low and high levels leading to increased and decreased nuclear size, respectively, regardless of the type of lamin expressed (Jevtic et al., 2015). The phosphorylation of lamin B3 by protein kinase C plays a role in controlling nuclear size during early *X*. *laevis* development and in mammalian cells, indicating that this mechanism of nuclear size regulation is conserved (Edens and Levy, 2014; Edens et al., 2017).

Nucleocytoplasmic transport is crucial for nuclear size regulation, as increased nuclear influx and inhibited efflux are associated with nuclear volume expansion and blebbing. In fission yeast, excessive accumulation of mRNA and protein within the nucleus results in enlarged nuclear size (Kume et al., 2017). Factors that regulate nuclear import, such as Importin alpha and Nuclear Transport Factor 2 (NTF2), play a more significant role in determining nuclear size than the amount of DNA (Figure 1), perhaps by regulating the amount of nuclear lamins that are imported (Levy and Heald, 2010). Radial growth phase primary melanoma cells exhibit larger nuclei than normal melanocytes, in particular when NTF2 levels are reduced. In patient-derived melanoma cells, increasing NTF2 expression leads to reduced nuclear size, decreased cell motility and proliferation, and enhanced apoptosis, implicating NTF2 as a melanoma tumor suppressor (Vukovic et al., 2021).

The nucleoporin ELYS plays a vital role in the assembly of the NPC following mitosis. In mammalian cells, a reduction in ELYS levels results in fewer NPCs, impaired nuclear import, diminished localization of nuclear lamin B2, and smaller nuclei. Some of these effects can be rescued by enhancing nuclear import through the overexpression of Importin alpha. Conversely, ELYS overexpression leads to a higher density of NPCs, increased import of nuclear lamin B2, and larger nuclei (Jevtic et al., 2019). In other work, mutations that lead to the clustering and/or misplacement of NPCs result in changes to the shape of the nucleus (Cohen et al., 2003; Tamura and Hara-Nishimura, 2011; Joseph-Strauss et al., 2012). While these experiments show a correlation between NPC number/distribution and nuclear size, other studies suggest that NPC assembly and nuclear expansion are independently regulated (Ryan et al., 2003; Doucet et al., 2010; Titus et al., 2010; McCloskey et al., 2018).

## 3 Chromatin and epigenetics as regulators of nuclear morphology

In the following sections, we discuss how chromatin structure and epigenetic determinants affect nuclear morphology and, if known, cell and organismal function.

### 3.1 Epigenetic factors, histones, and nuclear lamins

Epigenetic regulators affect nuclear dimensions and are linked to abnormal nuclear shape (Imbalzano et al., 2013; Furusawa et al., 2015; Schreiner et al., 2015; Stephens et al., 2017; Senigagliesi et al., 2019; Stephens et al., 2019a). For instance, overexpression of the histone acetyltransferase BRD4 leads to enlarged nuclei in HeLa cells (Devaiah et al., 2016), and various chromatin components, including core histones, impact nuclear shape in MCF10A cells (Tamashunas et al., 2020). Depletion of the linker histone H1.0 was found to alter nuclear shape as measured using the elliptical Fourier coefficient ratio (Tamashunas et al., 2020). In hTERT-immortalized fibroblasts, expression of disease-relevant histone H3.3 mutations (e.g., K9M, K27M, K36M) caused nuclear shape abnormalities, including reduced nuclear size, decreased nuclear circularity, and a general increase in nuclear morphology variability across cells. These observed effects were not due to changes in cell number, toxicity, or lamin A localization (Schibler et al., 2023).

Interactions between chromatin and lamins further shape nuclear architecture (Karoutas et al., 2019; Stephens et al., 2019a). Studies of nuclear mechanics showed that chromatin mediates responses to minor deformations whereas lamin A/C responds to greater forces (Stephens et al., 2017). Modifying the levels of euchromatin and heterochromatin influences nuclear structure and stiffness (Stephens et al., 2018; Stephens et al., 2019a), with increased heterochromatin reducing nuclear blebbing in compromised nuclei (Stephens et al., 2019a; Stephens et al., 2019b). The absence of the acetyltransferase MOF or its binding partners alters nuclear mechanics, associated with reduced lamin acetylation and epigenetic changes (Karoutas et al., 2019). These observations highlight that nuclear size and shape are the result of intricate interactions between chromatin and nuclear structural proteins.

### 3.2 Lamin-independent effects

Chromatin is critical for the ability of the nucleus to withstand and respond to mechanical force (Reddy et al., 2008; Stephens et al., 2019a). Histone modifications are known to influence chromatin structure and nuclear morphology (Figure 2). Treating mammalian cells with histone deacetylase inhibitors to enhance euchromatin softens the nucleus, increasing blebbing (Stephens et al., 2018; Kalinin et al., 2021). Conversely, histone demethylase inhibitors that increase the amount of heterochromatin stiffen the nucleus, reducing blebbing (Stephens et al., 2018). In both scenarios, nuclear morphological changes occur independently of lamin alterations.

Although lamin disruptions are typically associated with nuclear blebbing (i.e., protrusions larger than 1 µm), changes in chromatin alone can lead to bleb formation without altered lamin levels. Mouse embryonic fibroblasts (MEFs) treated with a histone deacetylase inhibitor showed increased euchromatic H3K9ac and nuclear blebbing while lamin B1 and A/C levels remained unchanged (Stephens et al., 2017). Thus, chromatin decompaction alone can trigger nuclear blebbing without lamin depletion (Stephens et al., 2018). Contrary to studies linking nuclear blebbing with lamin B absence (Shimi et al., 2008), blebs induced with valproic acid (VPA) retained lamin B1 and A/C in 50% of treated MEF cells and 30% of treated HT1080 cells. Similar lamin B1 retention was observed in nuclear blebs when cells were treated with trichostatin A or 3-Deazaneplanocin A (DZNep) (Miranda et al., 2009; Stephens et al., 2017; Stephens et al., 2018; Stephens et al., 2019b; Esmaeili et al., 2020), suggesting nuclear blebbing can occur as a result of chromatin-mediated reductions in nuclear rigidity without nuclear lamina disruptions (Stephens et al., 2018).

### 3.3 Chromatin structure and DNA amount

An emerging viewpoint is that mechanical properties of the nucleus can influence nuclear morphology (Dahl et al., 2008; Stephens et al., 2019b). Alterations in chromatin modifications and spatial arrangement are linked to changes in nuclear shape (Stephens et al., 2018; Stephens et al., 2019a; Heijo et al., 2020; Flores et al., 2021). Elevating heterochromatin levels with histone demethylase inhibitors leads to increased chromatin stiffness that can rectify abnormal nuclear shapes (Stephens et al., 2018). As the nucleus expands, condensing chromatin takes up a smaller fraction of the nuclear space and increasing nuclear histone levels through the histone chaperone Npm2 further encourages nuclear enlargement (Chen et al., 2019). Conversely, supplementing *Xenopus egg* extract with the histone methyltransferase Set9 (Batista and Helguero, 2018) or DNA methyltransferase inhibitor Zebularine (Yoo et al., 2004) increased the proportion of nuclear space filled by chromatin, with Set9 elevating H3K4me1 and Zebularine reducing 5-methylcytosine levels. These nuclei showed diminished growth and smaller final sizes, further highlighting how chromatin structure can affect nuclear size (Chen et al., 2024).

Nuclei formed in *Xenopus* egg extract almost completely stopped growing when treated with DNA degrading benzonase, regardless of the nuclear size when treatment was initiated (Chen et al., 2024). Nuclear F-actin plays a role in the expansion of the nucleus (Baarlink et al., 2017; Huang et al., 2022) but benzonase-treated nuclei formed in extracts with intact actin still failed to expand. These findings suggest that DNA is crucial for nuclear growth, even in the presence of F-actin (Chen et al., 2024). Massive increases in DNA content can affect nuclear size as nuclei assembled in *X*. *laevis* egg extract using axolotl sperm chromatin, which contains 20-fold more DNA than *Xenopus*, exhibited a doubling in nuclear cross-sectional area (Chen et al., 2024). This indicates that nuclear size can be responsive to DNA quantity (Levy and Heald, 2010; Heijo et al., 2020). When transcriptionally inert *Xenopus* egg extracts were treated with VPA to increase H3K9ac or DZNep to decrease H3K27me3, nuclei were smaller without any change in nuclear import rate. Conversely, increasing H3K27me3 with the histone demethylase inhibitor Methylstat or increasing 5-methylcytosine levels with the DNA methylator NDMA led to significant nuclear enlargement. This study showed that even with similar nuclear import rates, altered chromatin structure can reduce or increase nuclear growth in a transcription-independent manner (Chen et al., 2024).

### 3.4 Cellular mechanics and mechanosensation

The intermediate filament protein Keratin 17 can be found within the nucleus, interacting with proteins that play a role in organizing chromatin. Keratin 17 knockout in tumor-derived HeLa and A431 cell lines leads to a decrease in histone methylation and acetylation with a concomitant increase in nuclear size (Jacob et al., 2020). Fibroblasts, the predominant cells within connective tissues, are subjected to substantial compressive forces from the surrounding extracellular matrix and fluid during activities like walking, sitting, and sleeping. Applying a static compressive force to cultured mouse fibroblasts increases nuclear levels of histone deacetylase 3, which promotes heterochromatin formation. Upon removal of the compressive force, the cells revert to their original chromatin condensation state (Damodaran et al., 2018). Whether nuclear morphology is affected under these conditions is an interesting area for future study.

Cell stretching activates mechanosensitive channels, triggering transient calcium influx (Kim et al., 2015) and increased chromatin compaction (Heo et al., 2015; Heo et al., 2016; Le et al., 2016). Mechano-transduction mediated by these channels can protect against abnormal nuclear morphology. For example, in VPA-treated MEFs that exhibit chromatin decompaction, increasing the extracellular concentration of magnesium chloride induced heterochromatin formation through histone methyltransferases, leading to a reduction in nuclear blebbing. These cells also showed increased short-extension nuclear spring constants with no change in long-extension stiffness (Stephens et al., 2019b). Interestingly, MEF cells co-treated with VPA and the transcription inhibitor alpha-amanitin also exhibited reduced nuclear blebbing (Berg et al., 2023). Treating SKOV3 cells with trichostatin A to increase histone acetylation led to chromatin decompaction and a reduction in extracellular vesicle production upon cellular compression (Toth et al., 2004).

### 3.5 DNA damage and nuclear blebs

One of the hallmarks of many human diseases is DNA damage. MEFs and HT1080 cells treated with the DNA damaging agents cisplatin and bleomycin exhibited increased nuclear blebbing independently of passage through mitosis. Furthermore, DNA damage-induced nuclear blebs proceeded to rupture at a >90% frequency (Stephens et al., 2019b; Eskndir et al., 2025). Disrupting DNA damage response pathways, including p53, Rb, and BRCA2, can also increase the frequency of nuclear rupture (Yang et al., 2017; Kovacs et al., 2023). Micromanipulation force measurements of isolated vimentin-null MEF nuclei revealed that DNA damage significantly reduces chromatin-based nuclear stiffness (Stephens et al., 2017; Dos Santos et al., 2021; Currey et al., 2022). Heterochromatin was reduced in response to DNA damage through activation of Ataxia-telangiectasia Mutated kinase (ATM), leading to chromatin softening, reduced nuclear rigidity, and increased nuclear blebbing and rupture (Ziv et al., 2006; Ayrapetov et al., 2014; Eskndir et al., 2025). Inhibiting ATM during DNA damage treatment rescued heterochromatin levels and restored nuclear mechanics, shape, and integrity (Eskndir et al., 2025). Defects in nuclear morphology and integrity caused by DNA damage exacerbate a cycle of nuclear dysfunction in both confined and unconfined cells (Chen et al., 2018; Xia et al., 2018; Stephens et al., 2019b; Pho et al., 2024). Furthermore, inhibition of actin contractions can rescue DNA damage-induced changes in nuclear morphology (Pho et al., 2024).

Nuclear blebs often exhibit a unique chromatin signature. In MEFs, HT1080 fibrosarcoma, and PC3 prostate cancer cell lines it was recently discovered that nuclear blebs tend to exhibit reduced DNA density but that bleb formation does not correlate with lamin B1 levels (Bunner et al., 2025). Interestingly, increased DNA damage in such blebbed nuclei was independent of rupture (Chu et al., 2025). Disrupted nuclear morphology, including blebs, is associated with multiple disorders, and chromatin structure within nuclear blebs is often altered (Pujadas Liwag et al., 2025). There is direct evidence that the chromatin methylation status affects nuclear morphology. Loss of H3K9me3 increases nuclear blebbing and rupture due to decreased nuclear rigidity, while loss of H3K9me2 decreases nuclear blebbing and rupture accompanied by increased nuclear rigidity and more compact chromocenters (Manning et al., 2025). Biological processes like transcription can also regulate nuclear morphology, for instance chemical inhibition of transcription can suppress nuclear bleb formation and rupture (Berg et al., 2023). Taken together, DNA damage and chromatin status are important determinants of nuclear blebbing and rupture.

### 3.6 Cell migration, differentiation, and senescence

Alterations in chromatin structure and nuclear shape contribute to cell migration. In tenocytes, mechanical stretch caused reduced levels of H3K27me3 and increased levels of H3K9ac and H3K27ac, which in turn led to changes in nuclear morphology independently of lamin A/C, promoting tenocyte migration (Xu et al., 2023). Additionally, inhibiting histone methyltransferases with small molecule inhibitors affected nuclear morphology and integrity, diminished wound closure efficiency, and impeded cellular migration (Forman et al., 2024). In chicken primordial germ cells, disruption of the transcription factor Oct4 led to a reduction in H3K27ac modifications on active chromatin regions and altered cell migration (Meng et al., 2022).

The human immune system depends on a variety of cell types to establish and maintain its surveillance capabilities. Nuclear lobulation, a characteristic of many human immune cells, is a result of increased NE deformability and increased chromatin-NE interactions (Olins et al., 2001). Neutrophils adopt malleable polymorphonuclear structures by halting chromatin loop extrusion, thus enabling them to migrate through narrow interstitial spaces (Patta et al., 2024). Short range genome reorganization favors neutrophil migration such that the nucleus can alter its shape without causing chromatin damage (Jacobson et al., 2018).

Cell differentiation is often associated with changes in nuclear stiffness and morphology. For example, nuclei in embryonic stem cells are deformable and become six times stiffer in their terminal stages of differentiation (Pajerowski et al., 2007). Furthermore, increases in cell size have been shown to contribute to cellular senescence through genome dilution (Neurohr et al., 2019; Lanz et al., 2022; Lanz et al., 2024). Given that nuclear size typically scales with cell size, an interesting area for future study is whether increased nuclear size might also contribute to the onset of senescence.

### 3.7 Epigenetics and disease

Epigenetic modifications profoundly affect gene expression, and aberrant modifications have been linked to various diseases like cancer, neurological disorders, cardiovascular diseases, and more recently COVID-19 syndrome (Table 1) (Vodovotz et al., 2020). How epigenetic changes give rise to disease is a broad topic that has been extensively reviewed (Gasser and Li, 2011; Cavalli and Heard, 2019; Lopez-Lopez, 2023; McCulley, 2024; Tollefsbol, 2024). Here we will only touch on a few illustrative examples. In cancers, extensive DNA methylation and histone deacetylation occur within tumor suppressor genes, which results in their silencing and ultimately exacerbates disease. Aberrant DNA methylation and histone demethylation have been shown to promote the epithelial-to-mesenchymal transition in breast cancer cells, causing the cancer to proliferate and metastasize (Serrano-Gomez et al., 2016). Breast tumors defective for ARID1A, a component of the chromatin remodeling complex involved in nucleosome sliding, exhibit altered recruitment of histone deacetylase 2 and uncontrolled cell growth (Xu et al., 2020). Aberrations in epigenetic mechanisms are not limited to breast cancer. In glioblastoma multiforme, a rare and incurable adult brain tumor, hypermethylation of DNA is found in genes that regulate the WNT, Frizzled, and Ras pathways (Horiguchi et al., 2003; Gotze et al., 2010; Lambiv et al., 2011), contributing to cell proliferation. Mutations in ATP-dependent chromatin modifiers, including SMARCB1, BRG1, and BRM, have been identified in primary tumors and tumor-derived cell lines, including rhabdoid tumors, chronic myeloid leukemia, lung cancer, and prostate cancer (Salvatore and Vandenplas, 2003; Cho et al., 2004).

**TABLE 1:** An outline of diseases that are associated with epigenetic alterations.

Disease	Epigenetic changes	References
COVID-19	DNA methylation, histone acetylation	Pinto et al. (2020), Vodovotz et al. (2020), Foolchand et al. (2022)
Breast cancer	DNA methylation, histone acetylation, histone methylation	Serrano-Gomez et al. (2016), Xu et al. (2020)
Glioblastoma multiforme	DNA hypermethylation	Horiguchi et al. (2003), Gotze et al. (2010), Lambiv et al. (2011)
Type II diabetes	DNA hypermethylation	Ling et al. (2008), Barres et al. (2009), Yang et al. (2012), Hall et al. (2013)
Schimke immune-osseous dysplasia	Mutated chromatin remodeler SMARCAL1	Boerkoel et al. (2002)
Pediatric glioblastoma multiforme, pediatric adreno cortical tumor	Mutation in chromatin remodeler ATRX1	Liu et al. (2012), Schwartzentruber et al. (2012), Dyer et al. (2017)
Alzheimer’s disease	DNA methylation	Bijarnia-Mahay et al. (2014), Santana et al. (2023)

Altered epigenetics are associated with other diseases in addition to cancer. Hutchinson-Gilford progeria syndrome is characterized by abnormal nuclear shape, and increasing heterochromatin can restore normal nuclear morphology in cell lines that model the disease and in patient-derived cells (Scaffidi and Misteli, 2005). Altered DNA methylation patterns are observed in pancreatic islets and adipose and muscle tissues of type 2 diabetic mice and patients (Ling et al., 2008; Barres et al., 2009). In pancreatic islets, hypermethylated DNA can be detected in genes that regulate insulin secretion (Yang et al., 2012; Hall et al., 2013). In Alzheimer’s, altered DNA methylation is observed in disease-associated genes like amyloid precursor protein, Apolipoprotein E, and Ankyrin 1 (Bijarnia-Mahay et al., 2014; Santana et al., 2023). Mutations in the ATRX chromatin remodeling complex cause several X-linked syndromes that manifest with facial dysmorphism, urogenital defects, and α-thalassemia (Gibbons and Higgs, 2000). Mutations in the chromatin remodeler SMARCAL1 are a known cause of Schimke immuno-osseous dysplasia, an autosomal recessive disorder associated with T-cell immunodeficiency, spondyloepiphyseal dysplasia, renal failure, and other symptoms (Boerkoel et al., 2002). Thus, normal cellular physiology depends on precise regulation of the epigenetic state of chromatin and proper function of chromatin-associated molecules including histones and chromatin modifiers. Open questions relate to whether epigenetics influence disease phenotypes through altered nuclear morphology.

## 4 Emerging techniques and models in the field of nuclear morphology

Over the past 150 years, there have been significant advancements since Sir Lionel Beale first observed alterations in typical nuclear structures in various diseases, including cancer. To this day, the Pap smear, introduced by George Papanicolaou, remains a diagnostic tool for detecting abnormal nuclear structures in cervical tissue samples (Flores et al., 2021). Microscopy serves as an essential means of examining the nuclear condition of cells, offering crucial insights into nuclear shape, nuclear mechanics, protein distribution, and genome arrangement (Kim and Lakadamyali, 2024). Researchers are beginning to elucidate the connections between mechanics and morphology by integrating atomic force microscopy with side-view light sheet microscopy. Offering high spatiotemporal resolution, this approach has been used to observe cells during compression, leading to empirical models for nuclear deformation applicable to atypical nuclear shapes (Hobson et al., 2020; Hobson and Stephens, 2020). RD-SPRITE explores the interactions between chromatin and RNA (Quinodoz et al., 2021). Spatial multi-omics, which combines FISH and immunofluorescence techniques, have been used to chart nuclear architecture, and *in situ* genome sequencing facilitates the simultaneous visualization and sequencing of the genome at the nucleotide level (Palihati and Saitoh, 2024). Microfluidic devices constructed from polydimethylsiloxane can be used to replicate physiological conditions for the study of intracellular mechanics and dynamics, mimicking conditions relevant to cancer cell invasion and immune cell recruitment (Davidson et al., 2015).

The intricate nature of cells and their organelles poses challenges in studying fundamental molecular processes within living organisms. One approach to addressing this issue is bottom-up synthetic biology, which involves creating life-like systems from molecular components (Blain and Szostak, 2014; Auslander et al., 2017; Gopfrich et al., 2018). This multidisciplinary area incorporates chemistry, biology, and engineering (Sismour and Benner, 2005). One of the challenges in bottom-up synthetic biology is to develop systems that mimic life and sustain physiochemical balance using a minimal set of components (Smigiel et al., 2019). A model system commonly used to study organelle size, morphology, and function is cell-free *Xenopus* egg extract. Nuclei assembled in *Xenopus* extract exhibit the typical structures and activities of nuclei found in living cells (Chen and Levy, 2018; Mishra and Levy, 2022b). The open biochemical nature of the system allows for the addition of recombinant proteins and removal of endogenous proteins by immunodepletion, as well as exogenous addition of small molecule inhibitors or activators without pleiotropic effects on transcription, translation, and cell cycle progression (Sengupta et al., 2025). Furthermore, fluorescently labeled proteins allow for live time-lapse microscopy of organelle dynamics (Jevtic and Levy, 2018). Lastly, high-throughput imaging for siRNA screening represents an effective approach to identify regulators of nuclear architecture in an unbiased manner (Schibler et al., 2023). Interestingly, this latter study identified a number of nuclear morphology effectors in two different cell types but there was very little overlap in the hits, highlighting unresolved questions about the complexity of nuclear morphology control.

## 5 Conclusion

How nuclear morphology is regulated is a fundamental question of cell biology which remains to be understood completely. This regulation is critical as numerous diseases present with abnormal nuclear phenotypes that are often used by clinicians for diagnostic purposes. Several factors have been identified as contributors to this regulation; however important knowledge gaps still exist. In this review, we have discussed some of the known regulators of nuclear morphology, with a particular focus on how chromatin influences the regulation of nuclear structure and function. Aberrations in epigenetic modifications are the underlying cause for numerous diseases (Farsetti et al., 2023). At the same time, changes in the epigenetic landscape also impact nuclear morphology and function. To what extent aberrant nuclear morphology, altered epigenetic status, and disease phenotype are linked is a critical open question. Simplified model systems like *Xenopus* egg extract and novel approaches like hydrogel chambers are being increasingly used to mimic physiological cellular conditions and advance our knowledge of fundamental processes like the regulation of nuclear morphology. At the same time, advanced microscopy techniques have improved our ability to visualize component molecules and structures relevant to epigenetics and nuclear morphology. The next decade promises to answer key questions at the intersection of chromatin biology, nuclear morphology, and disease.
